# Gut–kidney crosstalk in septic acute kidney injury

**DOI:** 10.1186/s13054-018-2040-y

**Published:** 2018-05-03

**Authors:** Jingxiao Zhang, Ghada Ankawi, Jian Sun, Kumar Digvijay, Yongjie Yin, Mitchell H. Rosner, Claudio Ronco

**Affiliations:** 1 Department of Emergency and Critical Care Medicine, The Second Hospital of Jilin University, Changchun, China; 2 Department of Internal Medicine and Nephrology, King Abdulaziz University, Jeddah, Saudi Arabia; 3 International Renal Research Institute of Vicenza (IRRIV), Vicenza, Italy; 4 Division of Nephrology, University of Virginia Health System, Charlottesville, VA USA; 5 Department of Nephrology, Dialysis and Transplantation, San Bortolo Hospital, Vicenza, Italy; 6 Department of Nephrology and Research, Sir Ganga Ram Hospital, New Delhi, India

**Keywords:** Sepsis, AKI, Septic AKI, Gut–kidney crosstalk, Inflammation

## Abstract

Sepsis is the leading cause of acute kidney injury (AKI) in the intensive care unit (ICU). Septic AKI is a complex and multifactorial process that is incompletely understood. During sepsis, the disruption of the mucus membrane barrier, a shift in intestinal microbial flora, and microbial translocation may lead to systemic inflammation, which further alters host immune and metabolic homeostasis. This altered homeostasis may promote and potentiate the development of AKI. As part of this vicious cycle, when AKI develops, the clearance of inflammatory mediators and metabolic products is decreased. This will lead to further gut injury and breakdown in mucous membrane barriers. Thus, changes in the gut during sepsis can initiate and propagate septic AKI. This deleterious gut–kidney crosstalk may be a potential target for therapeutic maneuvers. This review analyses the underlying mechanisms in gut–kidney crosstalk in septic AKI.

## Background

Acute kidney injury (AKI) is a serious complication in critically ill patients and its development is associated with a high rate of mortality and morbidity as well as increased costs [1]. Sepsis is the leading cause of AKI in the intensive care unit (ICU), and 45 to 70% of all AKI is associated with sepsis [2]. Dialysis requiring AKI, when associated with distant organ dysfunction such as cardiac or respiratory failure, is associated with mortality rates as high as 60 to 80% [3, 4]. Septic AKI is a complex and multifactorial process, and our understanding of its pathogenesis remains incomplete [5]. The current understanding of septic AKI involves: microcirculatory abnormalities, renal tubular epithelial cell metabolic dysfunction and injury, and inflammatory changes [6].

Inflammation is a prominent component of septic AKI. Sepsis and inflammation at the tissue and cellular levels are associated with decreased levels of intracellular adenosine triphosphate (ATP) and with mitochondrial injury in the kidney [7]. It has been well described that higher cytokine concentrations are associated with slower renal recovery from AKI and increased mortality rates [8, 9].

**Fig. 1:**
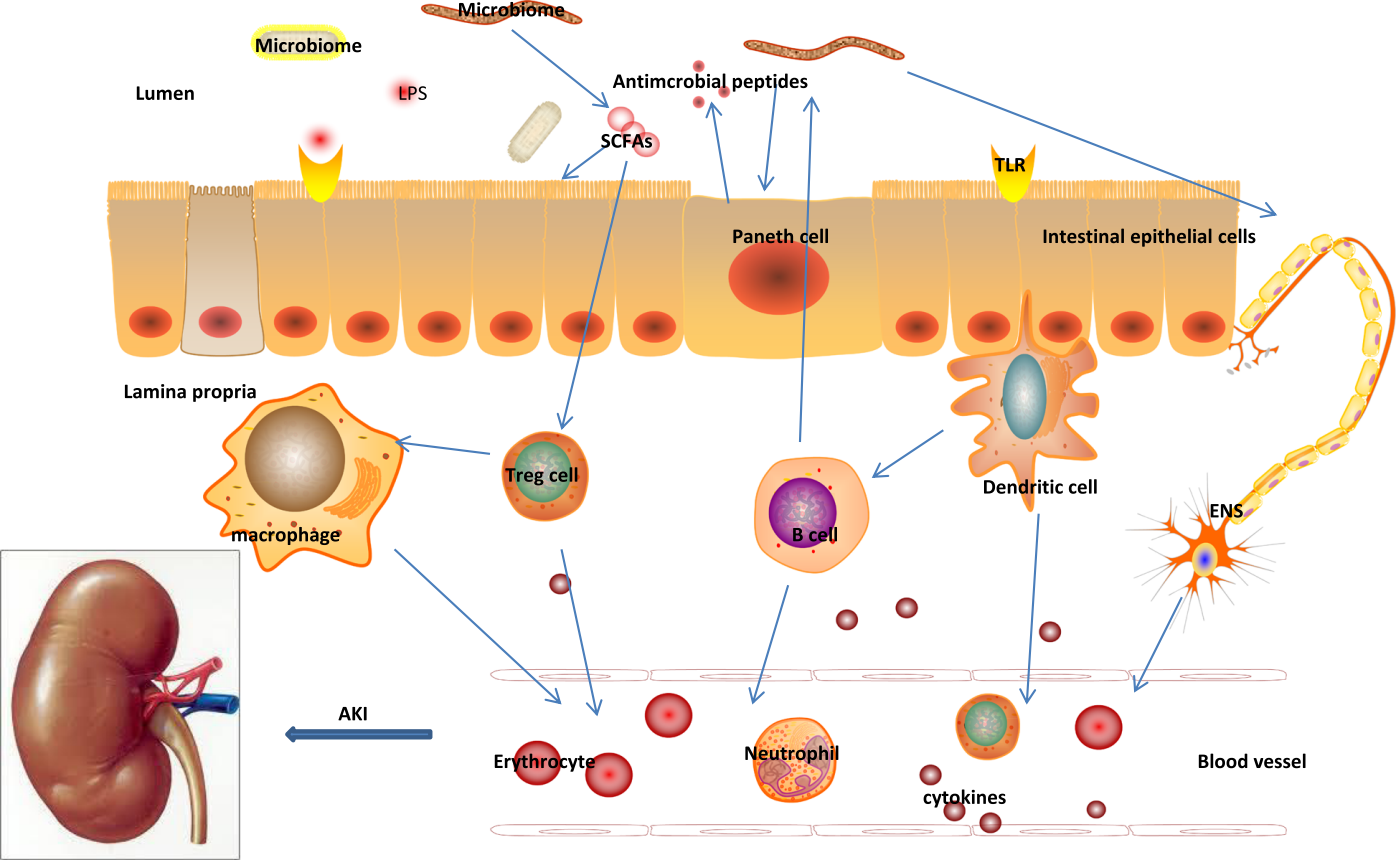
Effect of disruption of the gut mucosal barrier and bacterial translocation on the systemic inflammatory response in septic AKI. During sepsis, the combined effect of disruption of the mucus membrane barrier, a shift in the composition and virulence of intestinal microbes, and microbe translocation in gut lead to expansive inflammation, which will further alter host immune and metabolic homeostasis. The altered immune homeostasis and systemic inflammation can promote AKI in sepsis

The gastrointestinal tract (gut) is the most common source of secondary infections which occur predominately in the later stages of sepsis. The gut is home to 70–80% of the body’s immune cells and at least hundreds of different microbiome species [10]. In septic patients, inflammation and hypoperfusion play an important role in the pathophysiology of gut injury [11]. Gut injury may lead to impaired gut barrier function, which may result in translocation of bacteria and toxins from the intestinal lumen to the mesenteric lymph and systemic circulation. Subsequently, this bacterial translocation may amplify the systemic inflammatory response and contribute to multiple organ failure and death [12]. This dysregulated crosstalk between the gut’s epithelium, immune system, endogenous microflora, and kidney may lead to worsening of systemic inflammation and potentiation of AKI [13] (Fig. 1).

The understanding of the gut’s role in initiating septic AKI has led to potential novel therapeutic targets that are currently under investigations. In this review, we summarize the underlying mechanisms of gut–kidney crosstalk in septic AKI.

## The effect of septic AKI on gastrointestinal function

**Fig. 2:**
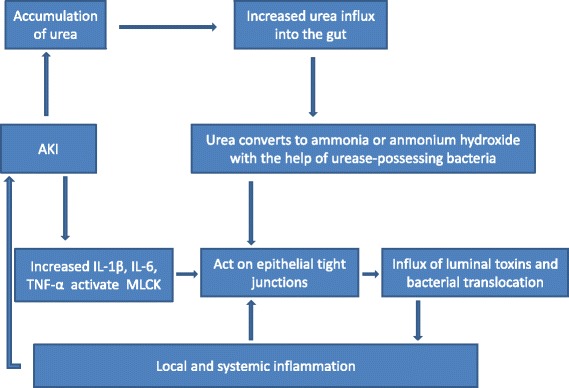
Effect of increased inflammatory cytokines and decreased urea clearance in septic AKI on the gut

During septic AKI, increased inflammatory cytokines as well as impairment in the clearance of water and metabolic products (urea in particular) can cause gut injury (Fig. 2). Specifically, increased inflammatory cytokines can damage gut barrier function and increase permeability. The gut’s barrier function is due to apical tight junctions and junctional adhesion molecules (JAM), which prevent luminal contents from escaping into the local extra-luminal environment. During septic AKI, increased levels of cytokines can act on these junctional complexes to modulate permeability [14]. Intestinal hyper-permeability may result when sepsis alters the expression of zonulaoccludens 1 (ZO-1), any one of multiple claudin isoforms, or occludin in the tight junction complex. Alternatively, hyper-permeability may be induced by altering expression of components of JAM [14]. Activation of myosin light chain kinase (MLCK) by cytokines can also worsen para-cellular permeability. MLCK phosphorylates myosin light chain, which results in contraction or opening of the apical tight junction [15]. MLCK activation is associated with an increase in interleukin (IL)-6, tumor necrosis factor α (TNF-α), and IL-1β. The net result is an increase in intestinal permeability. The increased intestinal permeability leads to an amplification of the systemic inflammatory response in a positive feedback response. The increased systemic inflammation further promotes kidney injury.

During septic AKI, the dysfunction in clearance of metabolic products and water can also directly weaken the gut barrier and increase gut permeability. In septic AKI, kidney dysfunction (with resulting retention of uremic solutes, urea, sodium, and water) as well as aggressive fluid resuscitation can cause a dramatic increase in gut wall edema. This can cause disruption of the colonic epithelial tight junction apparatus [16, 17]. Urea diffuses from the blood into the gut lumen and is metabolized by gut bacterial urease to ammonia (CO(NH_2_)_2_ + H_2_O → CO_2_ + 2NH_3_). The ammonia is converted into caustic ammonium hydroxide (NH_3_ + H_2_O → NH_4_OH), which is capable of disrupting tight junction proteins that seal the gap between epithelial cells [17]. Breakdown of this protein triggers influx of luminal toxins as well as bacterial translocation, thus promoting local and systemic inflammation and further damaging the gut’s barrier function.

### Increased cytokine levels in septic AKI can lead to dysregulation of gut stem cell proliferation/apoptosis

Toll-like receptors (TLRs) which act as pathogen receptors are expressed on various types of epithelial cells, including in the kidney and gut [13, 18]. Intestinal stem cells express TLR4, which regulates whether they proliferate or die by apoptosis [19]. On a cellular level, crypt proliferation is markedly decreased and both crypt and villus apoptosis are simultaneously increased following sepsis [20]. Although epithelial migration is slowed in critical illness in a TLR4-dependent manner, changes in proliferation and apoptosis overwhelm this slowed migration of cells, resulting in a marked diminution of villus length during sepsis [21]. Simultaneously, critical illness induces global alterations in the mucus layer (reduced thickness, diminished luminal coverage, and poor adherence) and altered gut barrier function [22]. Furthermore, increased cytokine concentrations seen in septic AKI can impair gut cell regeneration and stimulate apoptosis in a TLR4-dependent manner [23]. The increased inflammatory cytokine levels can also cause gut cell apoptosis directly. The net result is that gut apoptosis can enhance hyper-permeability, bacterial translocation, and expansion of the inflammatory response [24].

To summarize, during septic AKI, increased inflammatory cytokine concentrations and retention of urea as well as other metabolites and water can damage the gut barrier, leading to increased permeability. Impaired gut barrier function results in translocation of bacteria and toxins from the intestinal lumen to the mesenteric lymph system and to the systemic circulation. Subsequently, this bacterial translocation may amplify the systemic inflammatory response and contribute to multiple organ failure and death [12].

## The role of the microbiome in gut–kidney crosstalk

The intestinal microbiome is made up of more than 100 trillion microorganisms, and is continuously changing over the life of the host, based on diet, age, drug intake, and presence or absence of disease [25]. Increasingly, it is recognized that the microbiome plays a crucial role in the maintenance of health and that alterations in the type, number, and function of microorganisms in the microbiome can have a critical role on survival in critical illness [26]. During septic AKI, elevation in inflammatory cytokine levels as well as ischemia can induce changes in the constituent organisms that make up the microbiome of the gut. Importantly, the severity of inflammation can be modulated by the microbiome [25]. For instance, microbes or microbial products can actively change TLR expression in most cellular compartments of the gut tract, and this alters the host’s ability to sense and respond to the microbiota. Several other changes occur in gut physiology in septic patients, due to either extrinsic factors (antibiotics and parenteral nutrition) or intrinsic factors (systemic inflammation and gut leakage). These changes, in turn, influence the composition of the enteric flora [27]. A massive loss of microbe diversity occurs in patients with severe sepsis, particularly loss of anaerobic diversity [28]. For instance, within 6 h after the onset of sepsis, 90% of the normal anaerobic flora is lost in the gut [29]. In addition, critically ill septic patients relying on parenteral nutrition commonly have thinning of the protective mucus layer. This leads to a decrease in barrier integrity and the availability of immunomodulatory short-chain fatty acids (SCFAs) in the gut [13, 30].

Commensal bacteria (which are lost in sepsis) catabolize polysaccharides to generate SCFAs, and SCFAs play an important role in maintaining immune homoeostasis [31]. SCFAs can enhance the intestinal epithelial barrier function and activate the development of regulatory T (Treg) cells [31]. Monocytes are modulated by microbiota-derived products to prime natural killer (NK) cells via the interferon signaling pathway, which is pivotal in the immune response against other viral infections [32]. Interestingly and as a proof of the importance of the microbiome, therapy with three microbiota-derived SCFAs (acetate, propionate, and butyrate) can improve kidney function in a septic AKI model [32]. This protection was associated with low levels of local and systemic inflammation, oxidative cellular stress, cell infiltration/activation, and apoptosis. In addition, SCFAs ameliorated the effects of hypoxia in kidney epithelial cells by improving mitochondrial biogenesis.

## Effect of gut injury on the kidney in septic AKI

**Fig. 3:**
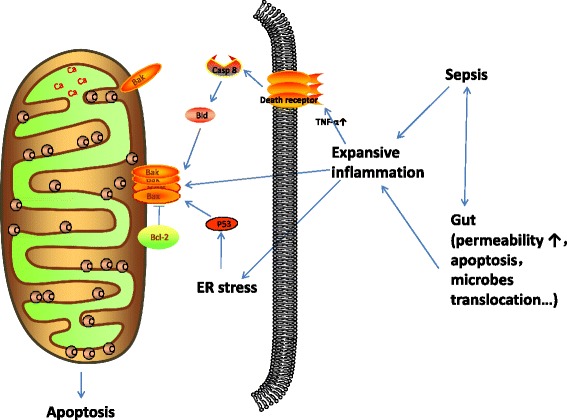
The different pathways (intrinsic and extrinsic) involved in renal apoptosis in septic AKI. *ER* endoplasmic reticulum

The impaired gut barrier function and bacterial translocation in septic AKI increase systemic inflammation, which are associated with slow renal recovery and mortality [8, 9]. After gut injury, the increased permeability results in translocation of bacteria and toxins from the intestinal lumen to the mesenteric lymph and systemic circulation [33, 34]. The influx of toxin contents can directly enter the circulation [35, 36] and/or educate circulating immune cells [37–39], which will cause an amplified inflammation in septic AKI that shifts metabolism towards aerobic glycolysis [40]. These changes are associated with decreased levels of intracellular adenosine triphosphate (ATP) and with mitochondrial injury in the kidney [7]. The decreased ATP synthesis and mitochondrial injury are the main causes of kidney dysfunction and injury [9]. The increased inflammatory cytokines (especially TNF-α) can induce kidney apoptosis through the extrinsic pathway in septic AKI [41, 42]. Up-regulation of Bcl-2 consistently blocks Bax and Bak activation, resulting in the preservation of mitochondrial integrity and cell viability and further supports the intrinsic pathway of apoptosis in septic AKI [41] (Fig. 3).

### The role of monocytes/macrophages in gut–kidney crosstalk

Monocytes/macrophages play an important role in the initiation or progression of inflammatory diseases [43]. Inflammation is closely related to macrophage activation: M1 macrophages exert pro-inflammatory activities, whereas M2 macrophages are involved in resolving inflammation [44] and in facilitating tissue repair [45]. Infection causes monocytes to migrate and infiltrate into organs where they differentiate via M1 or M2 pathways into pathogen-killing or tissue-repair phenotypes, respectively. Therefore, strategies that limit early macrophage infiltration or activation may represent a novel approach in the prevention or treatment of AKI in septic patients. However, the signaling pathway involved in the repair mechanism of M2 macrophages needs further investigation [46]. During sepsis, as the gut barrier function is impaired, bacteria translocation and expanding inflammation change the immune microenvironment, which may determine the fate of macrophage differentiation in the gut, kidney, or other distant organs into a more pro-inflammatory phenotype.

## Targeting the microbiome for therapeutic gain in gut–kidney crosstalk

As the gut plays an important role in the progression of inflammation and septic AKI, efforts to modulate the gut microbiome and barrier function to improve outcomes make great sense. Strategies include intake of live microbiota, addition of the necessary nutrients for microbiota regeneration, and administration of exogenous supplements such as SCFAs that are the products of microbiota. Selective decontamination of the digestive tract, probiotics, phosphate, and SCFAs are the most studied interventions in this regard and targeting the microbiome for therapeutic gain to regulate the immune function is a promising strategy to improve outcomes in sepsis. However, human data are still evolving in this therapeutic area.

### Selective decontamination of the digestive tract

Selective decontamination of the digestive tract (SDD) uses non-absorbable microbials, which are applied daily in the oropharynx and the gastrointestinal tract. The aim of this intervention is to prevent secondary colonization and overgrowth of potential bacterial pathogens while preserving the anaerobic microbiota, thereby preventing excess infectious disease in critically ill patients [47, 48]. As 90% of the normal anaerobic flora is lost after a sudden insult such as sepsis [29], the addition of SDD to a septic AKI patient is promising to rebuild the gut’s barrier, microbiome, and immune function. The aim of this intervention is to break down the continuous cycle of injury followed by amplification of inflammation that occurs in the gut–kidney crosstalk pathway [32]. A comprehensive systematic review and network meta-analysis [48], which encompasses more than 60 clinical studies and ten meta-analyses, suggested that SDD can prevent noscomial infections in critically ill patients and decreases overall mortality rates. However, randomized controlled trials are still needed in this area.

### Probiotics

Probiotics are live bacteria and yeasts that, when administered in adequate amounts, confer a health benefit on the host. Although their clinical use is rising, data on efficacy are still emerging. In a murine cecal ligation and perforation model of sepsis, the administration of the probiotics *Lactobacillus rhamnosus GG* (LGG) and *Bifidobacterium longum* (BL) improved mortality following sepsis and prevented sepsis-induced changes in gut epithelial apoptosis and proliferation [49]. Additionally, probiotics can attenuate growth of pathogenic intestinal bacteria, potentially limiting endotoxin production and preventing bacteremia [50]. As a corollary to probiotic administration, there is also increasing evidence that fecal microbiota transplantation is significantly more effective in the treatment of recurrent *Clostridium difficile* infection than standard antibiotic therapy by increasing fecal bacterial diversity in recipients [51].

### Short-chain fatty acids

**Fig. 4:**
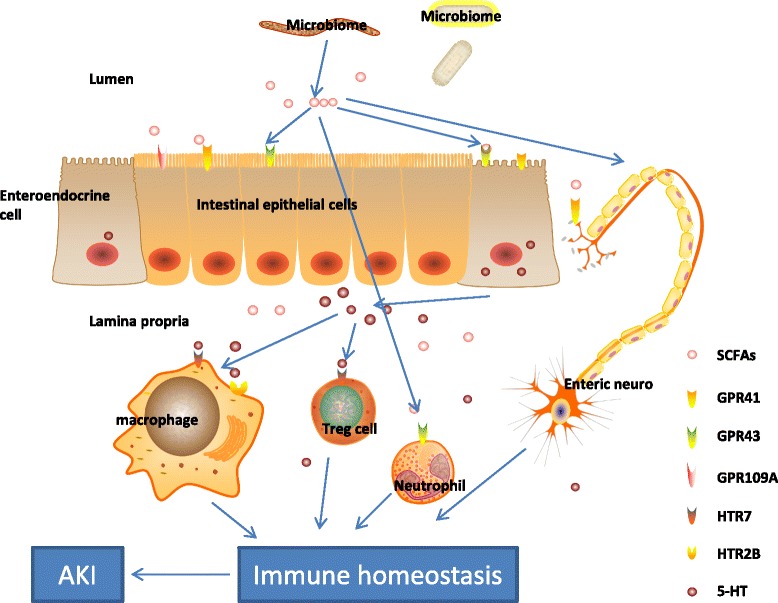
SCFAs, including acetate, propionate, and butyrate, are produced at high concentration by bacteria in the gut and subsequently released in the bloodstream. Receptors for SCFAs are G-protein-coupled receptors, GPR41, GPR43, GPR109A. Propionate is the most potent agonist for both GPR41 and GPR43. Acetate is more selective for GPR43, whereas butyrate and isobutyrate (nicotinic) are more active on GPR41. GPR41 is expressed in a number of tissues. GPR43 is selectively expressed in leukocytes and recruited toward sites of bacterial infection. Nicotinic acid can be anti-inflammatory in monocytes. Butyrate can also inhibit Jun NH(2)-terminal kinase activation and cytokine transcription in mast cells. SCFAs promote biosynthesis of 5-HT, enhancing T-cell activation by signaling through the 5-HT7 receptor, skews human macrophage polarization through HTR2B and HTR7, and activates immune responses and inflammation

SCFAs are microbial metabolic products of dietary fibers, and the most studied SCFAs are butyrate, propionate, and acetate. These metabolites are sensed by the G-protein-coupled receptors (GPR) on intestinal epithelium and can also diffuse across the epithelium [13, 30] to affect enteric nervous and immune systems. SCFAs can directly affect Treg cells [52, 53], neutrophils [54], monocytes [55], and mast cell [56] through the GPRs expressed on them, which will modulate the immune homeostasis of the body. It has been shown in animal experiments that therapy with three microbiota-derived SCFAs (acetate, propionate, and butyrate) improved renal dysfunction in a sepsis model, largely through epigenetic modulation of the inflammatory process [32]. SCFAs were also sufficient to up-regulate serotonin (5-HT) [57] . 5-HT can promote immune function of leukocytes by either enhancing dendritic cell-mediated T-cell activation or affecting macrophage polarization and phagocytosis [58] (Fig. 4). This promising avenue of research requires future study.

### Other therapies

The most common treatment for septic AKI is continuous renal replacement therapy (CRRT). CRRT can aid in removal of uremic toxins, sodium, and excessive volume. Thus, CRRT might positively impact gut wall edema and lessen the risk of bacterial translocation; future studies on CRRT should explore this possibility.

## Conclusions

Many aspects of sepsis remain undefined, and the interplay between the gut and kidney during septic AKI remains an interesting avenue for investigation. During sepsis, the combined effect of erosion of the mucus barrier, a shift in the composition and virulence of intestinal microbes, and the inability of the host epithelium to regulate its proliferative and apoptotic responses may lead to a tipping point in gut function where cascading inflammation drives AKI. During AKI, the clearance of inflammatory mediators is decreased, and metabolic products accumulate that can increase systemic inflammation. The continuous cycle of injury/amplification of inflammation can lead to devastating consequences. In theory, rational therapies aimed at restoring gut integrity, the microbiome, and the homeostatic balance between the two systems represents an exciting avenue in the battle against critical illness.

## Abbreviations

5-HT: Serotonin
AKI: Acute kidney injury
ATP: Adenosine triphosphate
Bcl-2: B-cell lymphoma-2
BL: *Bifidobacterium longum*
CRRT: Continuous renal replacement therapy
ENS: Enteric nervous system
GPR: G-protein-coupled receptors
ICU: Intensive care unit
IECs: Intestinal epithelial cells
IL: Interleukin
JAM: Junctional adhesion molecules
LGG: *Lactobacillus rhamnosus GG*
LPS: Lipopolysaccharide
MCP-1: Monocyte chemo attractant protein-1
MLCK: Myosin light chain kinase
mTOR: Mechanistic target of rapamycin
NK: Natural killer
PRR: Pattern recognition receptor
SCFA: Short-chain fatty acid
SDD: Selective decontamination of the digestive tract
TLRs: Toll-like receptors
TNF-α: Tumor necrosis factor α
Treg: Regulatory T
ZO-1: Zonulaoccludens 1
